# Patientensteuerung aus gesundheitsökonomischer Perspektive: Bedingungen wirksamer Kostenbeteiligung im europäischen Vergleich

**DOI:** 10.1007/s00103-026-04301-y

**Published:** 2026-09-21

**Authors:** Sebastian Böhme, Jessica Paas, Zoe Konjer, Jonas Meyrer, Jonas Schreyögg

**Affiliations:** Hamburg Center for Health Economics, Universität Hamburg, Esplanade 36, 20354 Hamburg, Deutschland

**Keywords:** Praxisgebühr, Fehlinanspruchnahme, Selbstbehalt, Verteilungswirkung, Primärarztsystem, Practice fee (Praxisgebühr), Inappropriate utilisation, Deductible, Distributional effects, Primary care gatekeeping

## Abstract

Steigende Gesundheitsausgaben und der demografische Wandel stellen die Finanzierung und Steuerung gesundheitlicher Versorgung in Deutschland vor zunehmende Herausforderungen. Zuzahlungen werden in der gesundheitspolitischen Debatte regelmäßig als Instrument zur Reduktion von Fehlinanspruchnahme diskutiert, zuletzt im Kontext eines Primärarztsystems. Der vorliegende Beitrag analysiert die institutionelle Ausgestaltung und empirische Evidenz finanzieller Steuerungsinstrumente in 4 europäischen Gesundheitssystemen (Deutschland, Niederlande, Frankreich, Dänemark) und führt die Befunde in einer gesundheitsökonomischen Synthese zusammen. Die Wirksamkeit von Zuzahlungen erweist sich als Funktion dreier ineinandergreifender Bedingungen: der Leistungsart, der verhaltensökonomischen Ausgestaltung und der institutionellen Architektur. Uniforme Zuzahlungen wirken regressiv und belasten Versicherte mit geringem Einkommen überproportional. Eine Wiedereinführung der Praxisgebühr in ihrer früheren Form ist auf Basis der internationalen Evidenz nicht zu empfehlen. Sinnvoll erscheint hingegen eine Konstruktion, die Zuzahlungen als gezieltes Lenkungsinstrument an jenen Stellen einsetzt, an denen Fehlinanspruchnahme empirisch festgestellt wird, etwa beim unangemessenen Verlassen eines koordinierten Versorgungspfades. Voraussetzung ist eine Ausgestaltung, die verhaltensökonomische Effekte und einkommensbezogene Unterschiede berücksichtigt. So konzipiert können Zuzahlungen zur Verbesserung der Patientensteuerung beitragen, ohne den Zugang zur Regelversorgung zu gefährden.

## Abstract (en)

Rising health expenditures and demographic change increasingly challenge the financing and steering of healthcare in Germany. Cost-sharing instruments are recurrently discussed as a means of reducing inappropriate utilisation, most recently in the context of a primary care gatekeeping system. This article analyses the institutional design and empirical evidence on financial steering instruments in four European health systems (Germany, the Netherlands, France, Denmark) and synthesises the findings from a health economics perspective. The effectiveness of copayments emerges as a function of three interrelated conditions: the type of service, the behavioural–economic design and the institutional architecture. Uniform copayments are regressive and disproportionately burden insured persons with low income. Based on the international evidence, a reintroduction of the previous German practice fee (Praxisgebühr) is not advisable. A more promising approach would design copayments as a targeted steering instrument at points where inappropriate utilisation is empirically identified, such as when patients inappropriately bypass a coordinated care pathway. This requires accounting for behavioural–economic effects and income-related differences. Configured in this way, copayments can contribute to improving patient steering without jeopardising access to standard care.

## Einleitung

Die gesetzliche Krankenversicherung (GKV) steht unter anhaltendem Finanzierungsdruck. Trotz steigender Beitragssätze ist die Differenz zwischen Einnahmen und Ausgaben in den vergangenen Jahren wiederholt gewachsen [1]. Parallel dazu werden in der gesundheitspolitischen Debatte regelmäßig nachfrageseitige Steuerungsinstrumente diskutiert, von der Wiedereinführung einer Praxisgebühr [2] über einkommensabhängige Selbstbehalte [3] bis hin zu Wahltarifen mit geringeren Beiträgen [2]. Gemeinsam ist diesen Vorschlägen die Grundidee, dass eine stärkere finanzielle Beteiligung der Versicherten an den Kosten ihrer Inanspruchnahme dazu beitragen kann, die Nachfrage nach Gesundheitsleistungen effizienter zu gestalten. Die politische Debatte um diese Instrumente bewegt sich in Deutschland häufig zwischen der Sorge um das Solidarprinzip und dem Ruf nach mehr Eigenverantwortung [4]. Eigenverantwortung ist in diesem Kontext nicht als Verantwortung für den eigenen Gesundheitszustand zu verstehen, sondern als Mitverantwortung der Versicherten für die gemeinsam getragenen Ressourcen. Im Sinne des Solidarprinzips funktioniert dies nur, wenn die Inanspruchnahme dieser nicht von ihren Kosten entkoppelt ist. Auf Basis der gesundheitsökonomischen Evidenz zu den Steuerungswirkungen von Kostenbeteiligungen möchte der vorliegende Artikel einen Beitrag zu dieser Debatte leisten.

Die gesundheitsökonomische Theorie bietet für diese Diskussion einen klaren analytischen Ausgangspunkt. Zuzahlungen adressieren ein Phänomen, welches bei der Inanspruchnahme von (medizinischen) Leistungen durch Versicherte auftritt und in der ökonomischen Literatur als moralisches Risiko (Moral Hazard) oder Third-Party-Payer-Effekt beschrieben wird. Moralisches Risiko bezeichnet die Verhaltensänderung von Versicherten infolge eines Versicherungsschutzes. Da die Kosten einer Inanspruchnahme überwiegend durch Dritte – hier die gesetzliche Krankenkasse – und nicht oder nur teilweise durch die Versicherten selbst getragen werden, werden Leistungen tendenziell häufiger nachgefragt als ohne Versicherung. Bereits Arrow (1963) und Pauly (1982) haben darauf hingewiesen, dass Krankenversicherungsschutz die Grenzkosten der Inanspruchnahme für Versicherte senkt und damit Anreize für eine über das effiziente Maß hinausgehende Nachfrage schaffen kann [5, 6]. Zuzahlungen, Selbstbehalte und andere Formen der Kostenbeteiligung setzen an diesem Problem an, indem sie die Preissensitivität der Versicherten gegenüber Gesundheitsleistungen (wieder‑)herstellen.

Das RAND Health Insurance Experiment (HIE) ist das bis heute einflussreichste experimentelle Forschungsprojekt zu Kostenbeteiligungen im Gesundheitswesen [7]. In diesem in den 1970er-Jahren in den USA durchgeführten Feldexperiment wurden Versicherte randomisiert Versicherungsplänen mit unterschiedlichem Selbstbehalt zugewiesen, wodurch sich die Wirkung von Kostenbeteiligungen auf die Inanspruchnahme erstmals kausal isolieren ließ. Es zeigte, dass Kostenbeteiligungen die Preiselastizität der Nachfrage beeinflussen. Die Preiselastizität beschreibt, wie stark die Inanspruchnahme einer Leistung auf eine Preisänderung reagiert. Die Einführung einer Kostenbeteiligung setzt ein Preissignal für die medizinische Leistung, sodass der Preis die Inanspruchnahme beeinflussen kann. Entsprechend reduzierte sich die Inanspruchnahme medizinischer Leistungen im HIE in den Gruppen mit Kostenbeteiligung signifikant. Allerdings reduzierten sich medizinisch notwendige und nicht notwendige Leistungen in ähnlichem Umfang, sodass eine zielgenaue Steuerung über das preisliche Signal allein nur begrenzt möglich erscheint. Die Kostenbeteiligung schadete gerade den vulnerabelsten Gruppen, da keine Befreiungsregelung existierte. Zudem zeigt die aktuelle Forschung zu Gesundheitskompetenz, dass es vielen Versicherten schwerfällt, zwischen notwendiger und unnötiger Inanspruchnahme zu differenzieren und im Gesundheitssystem kompetent zu navigieren [8].

Die verhaltensökonomische Forschung ergänzt diese Befunde um eine weitere Dimension. Menschen reagieren auf Kostenbeteiligungen nicht allein über rationale Preiskalküle, sondern werden in ihrem Entscheidungsverhalten maßgeblich durch Framing-Effekte, Verlustaversion und verzerrte Risikowahrnehmungen beeinflusst. So zeigen beispielsweise Hayen et al., dass eine Ausgestaltung als Verlust (z. B. Selbstbehalt) stärkere Nachfragereduktionen bewirkt als ein ökonomisch äquivalenter Gewinn (z. B. No-Claim-Bonus; [9]). Ein Befund, der verdeutlicht, dass die Wirksamkeit finanzieller Steuerungsinstrumente nicht nur von ihrer Höhe, sondern wesentlich von ihrer konkreten Ausgestaltung abhängt.

Die Literatur zeigt, dass Kostenbeteiligungen die Inanspruchnahme von Gesundheitsleistungen verändern. Die entscheidende Frage ist jedoch, unter welchen institutionellen Bedingungen sie zu einer tatsächlich verbesserten Patientensteuerung beitragen, ohne den Zugang für vulnerable Gruppen unverhältnismäßig einzuschränken. Europäische Gesundheitssysteme haben hierauf unterschiedliche Antworten gefunden. Für den vorliegenden Vergleich wurden Frankreich, die Niederlande und Dänemark ausgewählt. Die 3 Länder repräsentieren unterschiedliche institutionelle Ansätze nachfrageseitiger Steuerung in jeweils spezifischen Systemkontexten.

Frankreich nutzt wie Deutschland eine an die Erwerbsarbeit gekoppelte Sozialversicherung mit einkommensabhängigen Beiträgen, hat sich jedoch im Laufe der vergangenen Jahrzehnte zu einem gemischten System entwickelt, in dem steuerfinanzierte Elemente eine bedeutende Rolle spielen [10]. Die Kostenbeteiligung erfolgt überwiegend über prozentuale Zuzahlung ohne generelle Belastungsobergrenze und wird durch ein nahezu flächendeckendes System privater Zusatzversicherungen abgefedert.

Die Niederlande wiederum haben 2006 mit dem *Zorgverzekeringswet* (niederländisches Krankenversicherungsgesetz) die Trennung zwischen gesetzlicher und privater Krankenversicherung aufgehoben und alle Einwohner:innen in ein obligatorisches Versicherungssystem mit reguliertem Wettbewerb überführt [11]. Damit stehen die Niederlande exemplarisch für ein Reformmodell, das in der deutschen Debatte um eine „Bürgerversicherung“ wiederkehrend als Bezugspunkt herangezogen wird. Zentrales Steuerungsinstrument ist ein jährlicher Selbstbehalt, das sogenannte *Eigen Risico*.

Dänemark schließlich repräsentiert ein steuerfinanziertes Gesundheitssystem, in dem die Patientensteuerung in der Regelversorgung vollständig ohne nachfrageseitige Preisanreize organisiert ist. Versicherte haben universellen Zugang zu weitgehend kostenfreier Versorgung, jedoch mit einer strikten Gatekeeping-Funktion der Hausärzt:innen. Eine Umgehung dieser Gatekeeping-Struktur ist nur durch die Wahl eines Tarifes mit Eigenbeteiligung an den Mehrkosten direkter Spezialfacharztkonsultationen möglich, was nur von etwa 1 % der Bevölkerung genutzt wird [12].

Die Auswahl der Länder spannt damit ein Spektrum im Vergleich zum deutschen System auf, das von der Ausgestaltungsvariante unter systemverwandten Bedingungen (Frankreich) über eine institutionelle Reformalternative (Niederlande) bis zu einem strukturellen Gegenpol reicht, der Patientensteuerung ohne preisliches Signal in der Regelversorgung organisiert (Dänemark).

Die Ausgestaltung und Wirkung finanzieller Steuerungsinstrumente in diesen 3 Ländern werden aus gesundheitsökonomischer Perspektive analysiert und abschließend deren Übertragbarkeit auf das deutsche System diskutiert.

## Instrumente der Kostenbeteiligung in 4 europäischen Gesundheitssystemen

Aufgrund der verschiedenen Strukturen der Gesundheitssysteme der ausgewählten Länder werden unterschiedliche Instrumente der Kostenbeteiligung eingesetzt. Im folgenden Abschnitt werden die Gesundheitssysteme in den Niederlanden, Frankreich und Dänemark grundlegend beschrieben. In einer zusammenfassenden Tab. 1 werden die in diesen Ländern und im deutschen Gesundheitssystem eingesetzten Instrumente zur Kostenbeteiligung dargestellt. Diese lassen sich nach Zuzahlungen (pauschal und prozentual), Selbstbehalt (verpflichtend und freiwillig), Zusatzversicherungen sowie Regelungen zur Befreiung von Kostenbeteiligungen zum Schutz vulnerabler Gruppen unterteilen. Im anschließenden Abschnitt erfolgt eine länderspezifische Analyse der empirischen Evidenz hinsichtlich der Wirksamkeit der Instrumente auf die Patientensteuerung.

**Tab. 1:** Kostenbeteiligung im europäischen Vergleich (Deutschland, Niederlande, Dänemark und Frankreich)

	Deutschland (DE; [13])	Niederlande (NL; [11])	Dänemark (DK; [12])	Frankreich (FR; [10])
Zusatzversicherungen	Ja (ca. 50 %) Für Zahnmedizin, ambulante Zusatzleistungen, stationäre Wahlleistungen	Ja (ca. 90 %) Für z. B. Zahnmedizin, Physiotherapie, Hilfsmittel	Ja (ca. 35 %) Für z. B. Zahnmedizin, Physiotherapie, Hilfsmittel	Ja (= *Mutuelle; ca.* 95 %) Für *Ticket Modérateur* und z. B. Zahnmedizin, Hilfsmittel
Obligatorischer jährlicher Selbstbehalt	Nein	Ja *(= Eigen Risico* 385 € – 2026) Für Großteil ambulanter Leistungen (Ausnahme: Hausarztbesuche)	Nein	Nein
Freiwilliger (zusätzlicher) Selbstbehalt	Ja Wahltarife Krankenkassen (max. 600 € Beitragsrückerstattung)	Ja *Voluntary Deductible* (VD; bis max. 885 €; Prämienrabatt)	Nein	Nein
Pauschale Zuzahlung	Ja z. B. 10 €/Tag stationär (max. 28 Tage/Jahr)	Nein	Selten z. B. Physiotherapie Zahnmedizin, ambulant verordnete Arzneimittel	Ja z. B. 2 € für ambulante Leistungen, 20 €/Tag stationär, 1 €/Arzneimittelpackung
Prozentuale Zuzahlung	Ja z. B. Rezeptgebühr: 10 % (min. 5 €, max. 10 €)	Nein	Nein	Ja *Ticket Modérateur*: 30 % ambulante Leistung, 20 % stationäre Leistung, 0–100 % Arzneimittel (je Nutzen) *Balance Billing: *Zusatzhonorar für bestimmte ärztliche Leistungen/Hilfsmittel
Staffel‑/Deckelungslogik (Caps, Limits)	Ja Jahresbelastungsgrenze (2 % Bruttoeinkommen bzw. 1 % bei chronisch Kranken)	Ja *Eigen-Risico*-Cap pro Jahr (Kinder < 18 befreit); Einkommenszuschuss für Low-Income (*Zorgtoeslag*)	Ja Arzneimittel: Rückerstattung je Ausgabenhöhe (bis zu 4850 DKK); chronisch Kranke und Kinder befreit	Ja Kostenübernahme bei chronischen Erkrankungen; Zuschüsse Zusatzversicherung für Low-Income

Das niederländische Gesundheitssystem [11] ist als hybrides System von einer Krankenversicherungsstruktur nach dem Solidarprinzip und einem regulierten Wettbewerb privater Akteure geprägt. Die Bevölkerung ist verpflichtet, eine Krankenversicherung abzuschließen. Folgende Zuzahlungsmodalitäten sind mit dem Ziel der Patientensteuerung in den Niederlanden Teil des Versicherungssystems: ein festgelegter Selbstbehalt von jährlich 385 € für die Inanspruchnahme von Gesundheitsleistungen sowie Zuzahlungen für weitere medizinische Leistungen und Hilfsmittel.

Grundsätzlich gilt in Frankreich ein gemischtes System, das sich strukturell als soziales und finanziell durch ein staatliches Krankenversicherungssystem mit nationaler Solidarität definiert. In Frankreich sind alle Personen mit festem Wohnsitz oder Anstellungsverhältnis (mind. 3 Monate) in der Krankenversicherung versichert. Das Kostenbeteiligungssystem in Frankreich zur Steuerung der Versicherten ist durch Zuzahlungen für alle essenziellen Leistungen und ein flächendeckendes System privater Zusatzversicherungen geprägt [10].

Das dänische Gesundheitssystem [12] ist zum größten Teil durch Steuereinnahmen in einem universellen Wohlfahrtsmodell finanziert. Denn alle in Dänemark registrierten Bewohner:innen sind in der steuerfinanzierten Krankenversicherung eingeschlossen. Sie haben Anspruch auf ein umfangreiches Leistungspaket mit ambulanten, stationären und pharmazeutischen Gesundheitsleistungen, die zum Zeitpunkt der Inanspruchnahme weitgehend kostenlos sind und durch eine starke Gatekeeping-Struktur koordiniert werden. Versicherte können seit 1973 zwischen 2 Versicherungsoptionen wählen, dabei können sie sich für einen Tarif entscheiden, bei dem sie sich für einen direkten Zugang zu einem/r Fachärzt:in an den entstandenen Mehrkosten beteiligen müssen: In Gruppe 1 (ca. 99 % der Bevölkerung) sind Versicherte bei einem/einer Hausärzt:in eingeschrieben, der/die als Gatekeeper:in für den Zugang zu Spezialfachärzt:innen und Krankenhausbehandlungen fungiert. In Gruppe 2 (ca. 1 %) besteht direkter Zugang zu Spezialfachärzt:innen ohne Überweisung, allerdings beteiligen sich die Versicherten an den dadurch entstehenden Mehrkosten. Unabhängig davon sind jedoch auch in Dänemark von allen Versicherten Zuzahlungen für ambulante Arzneimittel und zahnärztliche oder physiotherapeutische Behandlungen zu leisten, die auf eine Steuerung für bestimmte Leistungsbereiche zielen.

### Deutschland

#### Zuzahlungen.

Spätestens seit den 1980er-Jahren sind Kostenbeteiligungen für Gesundheitsleistungen Bestandteil der GKV. Seit 2004 (*GKV Modernisierungsgesetz*) existieren mehrere Zuzahlungsregelungen zur primären Steuerung der Versicherten, um insbesondere medizinisch nicht notwendige Inanspruchnahme zu reduzieren und damit die Nutzung von Versorgungsleistungen effizienter zu gestalten. Die Zuzahlungen für Arzneimittel machen mit ungefähr 50 % einen Großteil der *Out-of-pocket*-Zahlungen aus [14]. Empirische Studien zur Effektivität der Patientensteuerung zeigen, dass höhere Zuzahlungen zu Arzneimitteln zu einer reduzierten Inanspruchnahme von Leistungen führen können. Nach einer Gesundheitsreform im Jahr 1997, in der die Zuzahlung zu verschreibungspflichtigen Arzneimitteln um 200 % erhöht wurde, zeigte sich als indirekter Effekt ebenfalls eine Reduktion der Inanspruchnahme von Hausarztterminen um bis zu 10 % [15].

Allerdings wirken sich diese Effekte einer effizienteren Versorgung häufig nur auf bestimmte Personengruppen aus, weshalb die Gesamtsteuerungswirkung letztlich meist als gering eingestuft wird [16]. Eine quasiexperimentelle Studie zeigt, dass multimorbide Personen oder Personen mit geringerem Einkommen von den Zuzahlungen überproportional stark betroffen sind [17]. Krankheitsspezifische Analysen, wie bei einer rheumatoiden Arthritis, verdeutlichen eine hohe finanzielle Belastung mit ca. 417 € pro Jahr, wobei die Arzneimittel ein Viertel des Betrags ausmachen [18].

#### Praxisgebühr.

Mit dem GKV Modernisierungsgesetz wurde 2004 zudem eine Praxisgebühr als explizites Steuerungsinstrument zur Reduktion von Arztkontakten eingeführt. Versicherte mussten einmal pro Quartal beim Termin in der Praxis 10 € zahlen [19]. Empirisch zeigte sich dadurch eine kurzfristige Reduktion der Anzahl der Arztbesuche von 4–8 %, insbesondere bei der jüngeren Bevölkerung [20]. Zudem war der Rückgang der Inanspruchnahme bei Personen mit niedrigem sozioökonomischen Status, geringerem Einkommen und geringerer Schwere der Erkrankung stärker ausgeprägt [21, 22]. Außerdem wurden kurzfristige Verschiebungen in der Art der in Anspruch genommenen Leistungen festgestellt: Es fanden weniger Primärkontakte bei Spezialfachärzt:innen statt, die Kontakte pro Fall pro Quartal bei chronisch erkrankten Versicherten stieg an und es wurden weniger Versicherte präventiv versorgt [23]. Schreyögg und Grabka zeigten, dass die Praxisgebühr chronisch Erkrankte und Personen mit geringem Einkommen nicht von einem Arztbesuch abhielt, was auf eine Effektivität der Befreiungsregelungen hindeutet [24].

In Untersuchungen auf Basis von Paneldaten über mehrere Jahre konnte jedoch kein Rückgang der Anzahl der Praxisbesuche gezeigt werden, sodass die Reform nicht die intendierten Effekte auf die Nachfrage der ambulanten Versorgung hatte [24–26]. Aufgrund der strukturellen Gegebenheit der Praxisgebühr – eine Zahlung pro Quartal – könnte es die Versicherten sogar eher angereizt haben, häufiger pro Quartal ihre:n Ärzt:in aufzusuchen [27]. Zum Ende des Jahres 2012 folgte daher die Abschaffung der Praxisgebühr. Im Nachgang zeigte eine Kohortenstudie, dass nach der Abschaffung die hausärztliche Koordinierungsfunktion abnahm [26].

#### Selbstbehalt.

Neben direkten, von den Versicherten zu leistenden Zuzahlungen existieren auch indirekte Formen der Kostenbeteiligung über Variationen in den Krankenkassentarifen, insbesondere Wahltarife, die im Gegenzug zur Zahlung eines Selbstbehalts eine Prämie auszahlen. Empirische Studien zeigen, dass Selbstbehaltstarife mit einer geringeren Inanspruchnahme von Gesundheitsleistungen einhergehen. So wurde eine signifikante Reduktion der Ausgaben für Krankenhausbehandlungen, Arzneimittel und Heilmittel um durchschnittlich 284 € gezeigt [28], während weitere Studien relative Rückgänge von bis zu 6 % zeigten [29].

Kostenbeteiligungen im deutschen Gesundheitssystem können durchaus zur Patientensteuerung beitragen. Sowohl für Arzneimittel als auch für Hausarztbesuche konnte eine preisinduzierte Reduktion der Inanspruchnahme gezeigt werden. Diese Instrumente setzen primär auf eine Steuerung der Nachfrage, ohne gezielt zwischen notwendiger und weniger notwendiger Inanspruchnahme zu differenzieren, was dazu führen kann, dass vulnerable Gruppen von den Auswirkungen betroffen sind und sich die intendierten Effekte nicht langfristig einstellen.

### Niederlande

#### Selbstbehalt.

Der Selbstbehalt bis zu einem Betrag von 385 € jährlich (Stand 2026) ist das zentrale Instrument der Kostenbeteiligung in den Niederlanden und zielt auf eine stetige, versorgungsübergreifende Steuerung ab. Das sogenannte *Eigen Risico* als Kernelement der Patientensteuerung zeigt in modellbasierten Schätzungen das Potenzial einer Reduktion der Gesundheitsausgaben von 6 % jährlich pro Erhöhung des Betrags um 100 € [30]. Dieses Potenzial wird auch im direkten Vergleich mit dem zuvor geltenden Modell der Beitragsrückerstattung bei Nichtinanspruchnahme von Leistungen deutlich. Die Versicherten reagierten auf die Einführung des Selbstbehalts mit einer um durchschnittlich 8,6 % stärkeren Reduktion ihrer jährlichen Gesundheitsausgaben als unter dem Beitragsrückerstattungsmodell [9]. In einer Difference-in-Differences-Analyse reduzierte ein zusätzlicher Selbstbehalt von 1 € die Gesundheitsausgaben im Vergleich zur Beitragsrückerstattung um durchschnittlich 0,18 €, dieser Effekt wurde besonders bei Personen aus Regionen mit geringerem Einkommen deutlich [31].

Der Selbstbehalt kann zusätzlich freiwillig im Gegenzug zu Prämienrabatten auf maximal 885 € erhöht werden (Art. 20 *Zorgverzekeringswet*). Empirische Evidenz zeigt, dass Wahlmodelle eines solchen freiwilligen Zusatz-Selbstbehalts (VD) für ca. 48 % der Versicherten finanziell vorteilhaft sein können, sich jedoch nur ca. 11 % dafür entscheiden [32]. Insbesondere gesunde, jüngere Männer mit niedrigen Vorjahreskosten profitierten von dem VD.

#### Zuzahlungen.

Im Jahr 2012 wurde die Zuzahlung zur fachärztlichen psychiatrischen Gesundheitsversorgung angehoben: Es zeigte sich eine deutliche Reduktion der Leitlinien-empfohlenen kontinuierlichen ambulanten Versorgung, während die akute psychiatrische Versorgung anstieg [33]. Lopes et al. fanden ebenfalls eine Reduktion der Wahrscheinlichkeit der Inanspruchnahme psychischer Gesundheitsversorgung um 13,6 % bei Frauen und 5,3 % bei Männern bei einer steigenden Zuzahlung mit Erreichen der Altersgrenze von 18 Jahren [34]. Diese höhere Kostenbeteiligung führte allerdings auch zu einer stärkeren Beeinträchtigung schwerkranker und einkommensschwacher Versicherter und konnte erhebliche Folgekosten verursachen, weshalb die Zuzahlung zur psychischen Gesundheitsversorgung in den Folgejahren wieder abgeschafft wurde [35].

Auch für Besuche in der Notaufnahme werden Zuzahlungen diskutiert. Befragungen zeigen, dass mehr als 70 % der sich selbsteinweisenden Notaufnahmepatient:innen bereit wären, für eine Behandlung in der Notaufnahme eine Zuzahlung zu leisten, wobei die Mehrheit einen Betrag zwischen 25 € und 50 € akzeptieren würde. Die akzeptierte Höhe der Zuzahlung hing jedoch maßgeblich vom Haushaltseinkommen, von der Dringlichkeit der Behandlung und vom Bildungsniveau ab [36].

Zusammenfassend zeigt das Kernelement im niederländischen Gesundheitssystem – das *Eigen Risico *– eine stärkere Steuerungsfunktion der Versicherten als Beitragsrückerstattungsmodelle zuvor [31]. Insbesondere die kontinuierliche Ausgestaltung des Selbstbehalts über die Versorgungsebenen hinweg verdeutlicht das Steuerungspotenzial. Außerdem existieren zusätzliche Versicherungsoptionen mit erhöhten Selbstbehalten, die besonders für junge, gesunde Personen mit geringen Leistungsausgaben vorteilhaft erscheinen.

### Frankreich

#### Zuzahlungen.

Zusätzlich zu dem zu zahlenden Beitrag zur Krankenversicherung gibt es in Frankreich Zuzahlungen, die von den Versicherten „out of pocket“ zu leisten sind (Tab. 1). Diese Ausgaben sind nicht übergreifend bis zu einem Gesamtbetrag gedeckelt, sondern versorgungsbereichsspezifisch. So müssen Versicherte u. a. in der ambulanten Versorgung und in der pharmazeutischen Versorgung jeweils maximal 50 € jährlich als feste Zuzahlung bezahlen [37]. Außerdem sind schwangere Frauen, Kinder und Personen mit mindestens einer von 32 festgelegten chronischen Krankheiten von krankheitsbezogenen variablen Zuzahlungen befreit [38]. Trotz dieser bestehenden Schutzmechanismen für die genannten Gruppen, sind vulnerable Gruppen von den höchsten Zuzahlungen betroffen [10]. Die finanzielle Belastung für sozioökonomisch schwächere Gruppen als Hürde im Zugang zur Versorgung wird häufig als Kritikpunkt genannt. Insbesondere weil es keine einheitliche, einkommensabhängige Obergrenze für Zuzahlungen gibt und finanzielle sowie administrative Hindernisse im Zugang zu einer hochwertigen Zusatzversicherung existieren, funktionieren die Schutzmechanismen nicht ausreichend [37, 39].

#### Zusatzversicherung.

Etwa 95 % der Bevölkerung versichern sich mit einer privaten Zusatzversicherung (*Mutuelle*) gegen die anfallenden variablen Zuzahlungen (*Ticket Modérateur;* [37]). Verschiedene Studien untersuchen, welche Faktoren die Entscheidung für eine Versicherung beeinflussen und welche Auswirkungen die Versicherung auf die Inanspruchnahme von Gesundheitsleistungen hat. Insbesondere das Einkommen beeinflusst die Wahl einer Zusatzkrankenversicherung. In der Bevölkerungsgruppe mit dem geringsten Einkommen haben über 11 % gar keine oder nur eine private Zusatzversicherung mit unzureichender Abdeckung von Leistungen [37]. Eine steigende Abdeckung durch die Versicherung führt jedoch im Gegenzug zu einer erhöhten Inanspruchnahme von Gesundheitsleistungen und dadurch zu steigenden Kosten [40].

Zuzahlungsmodalitäten in Frankreich können grundsätzlich eine Steuerungsfunktion der Versicherten übernehmen, werden jedoch durch Zusatzversicherungen und fehlende zielgerichtete Ausgestaltung in ihrer Wirksamkeit abgeschwächt. Sie wirken sich trotz bestehender Schutzmechanismen besonders auf vulnerable und einkommensschwächere Personengruppen als Zugangsbarriere aus.

### Dänemark

#### Zuzahlungen.

Für ambulant verordnete verschreibungspflichtige Arzneimittel fallen im dänischen Gesundheitssystem Zuzahlungen an. Diese sind gestaffelt und auf einen jährlichen Höchstbetrag von 4850 DKK (ca. 650 €) begrenzt [41]. Die Höhe der zu leistenden Zuzahlungen ist abhängig von der Morbidität: In einer populationsbasierten Registeranalyse (Stand 2020) zahlten Personen mit mehr als 5 chronischen Erkrankungen jährlich im Durchschnitt 320 €, Personen mit 2 chronischen Erkrankungen leisteten durchschnittlich 187 € während Personen ohne chronische Erkrankungen lediglich 44 € pro Jahr zahlten [42].

Empirische Studien zeigen, dass ein Anstieg der Zuzahlung für Arzneimittel in Dänemark um 100 € zu Reduktionen der Nachfrage von 2–7 % [43] bzw. 3,6–5 % [44] führte, besonders chronisch Kranke und ältere Menschen reagieren dabei deutlich weniger auf Preisänderungen.

Neben Zuzahlungen für Arzneimittel fallen auch für ambulante Behandlungen durch Psychotherapeut:innen prozentuale Zuzahlungen in Höhe von 40 % an. Eine Difference-in-Differences-Analyse zeigte, dass eine Abschaffung dieser Zuzahlung die Inanspruchnahme nahezu verdoppeln würde. Es wurde außerdem festgestellt, dass diese Veränderung leichte Anstiege der psychiatrischen Versorgung und der Verschreibung von Antidepressiva mit sich bringen würde [45].

In Dänemark existiert keine Zuzahlung für Besuche bei dem/der Hausärzt:in. Allerdings wurde vor einigen Jahren über eine Einführung einer Zuzahlung diskutiert [46]; ein politischer Konsens kam jedoch nicht zustande [47] und Stand 2024 gab es keine politischen Initiativen zur Einführung von Zuzahlungen für die ambulante Regelversorgung [12]. Dies passt zu Erkenntnissen einer Studie zur Zahlungsbereitschaft (Willingness to Pay, WTP), wonach mehr als 50 % der Bevölkerung eine Zuzahlung für Hausarztbesuche ablehnen. Ältere Personen > 65 Jahren und mit einem Einkommen > 200.000 DKK (≈26.800 €) wären grundsätzlich eher bereit eine Zuzahlung zu leisten, die in dieser Studie hypothetisch durchschnittlich bei 137 DKK (≈18,40 €) lag [48].

#### Zusatzversicherung.

Weiter kann eine freiwillige private Zusatzversicherung für die Übernahme der Zuzahlungen abgeschlossen werden. In Studien zeigte sich eine erhöhte Inanspruchnahme von Gesundheitsleistungen bei Personen mit dieser Zusatzversicherung im Vergleich zu Personen ohne Zusatzversicherung [49]. Die Wahrscheinlichkeit, über eine Zusatzversicherung zu verfügen, steigt mit dem Einkommen und der Dauer der schulischen sowie beruflichen Bildung. Bei Frauen ist diese Wahrscheinlichkeit höher als bei Männern und nimmt mit zunehmendem Alter bis zur Altersgrenze von 60 Jahren zu. Außerdem sind Personen mit schlechterem Gesundheitszustand seltener zusatzversichert. Daraus lässt sich schließen, dass die Verteilung der Mitglieder nach sozioökonomischen und gesundheitsbezogenen Merkmalen ungleich ist [50].

Die Möglichkeit, sich in Dänemark gegen Zuzahlungen versichern zu lassen, verringert die Steuerungsfunktion der Versicherten, da sich die Inanspruchnahme durch das Vorliegen der Zusatzversicherung erhöht. Bei bestimmten Leistungen wie der psychotherapeutischen Versorgung verdeutlicht eine reduzierte Inanspruchnahme die Steuerungsfunktion. Jedoch zeigt sich deutlich, dass Personengruppen mit geringem Einkommen oder mit schlechterem Gesundheitszustand unterschiedlich auf die Zuzahlungsmodalitäten reagieren. Insgesamt erfolgt die Steuerung in Dänemark stärker über institutionelle Mechanismen wie die Gatekeeper-Funktion als über finanzielle Anreize.

## Diskussion

In diesem Beitrag wurde zunächst das Steuerungsproblem skizziert, das entsteht, wenn Versicherte bei der Inanspruchnahme medizinischer Leistungen nur einen geringen oder keinen Teil der eigentlichen Behandlungskosten selbst tragen, und Zuzahlungen in ihren unterschiedlichen Ausgestaltungsformen als ein in europäischen Gesundheitssystemen verbreitetes Steuerungsinstrument identifiziert. In Deutschland werden rund 13 % der laufenden Gesundheitsausgaben unmittelbar von privaten Haushalten finanziert. Hierzu zählen gesetzliche Zuzahlungen ebenso wie weitere Eigenleistungen für Gesundheitsleistungen [51]. Dieser Anteil liegt in einer ähnlichen Größenordnung wie in Dänemark (12 %), den Niederlanden (13 %) und Frankreich (9 %; [10–12]).

Der Ländervergleich zeigt, dass Zuzahlungen in der Praxis mehrere Funktionen gleichzeitig erfüllen können. Wenn man der Unterscheidung von Robinson folgt, lassen sich 3 zentrale Begründungslinien unterscheiden [52]: Zuzahlungen sollen erstens die Inanspruchnahme steuern und einer durch Moral Hazard induzierten Überinanspruchnahme entgegenwirken. Sie können zweitens als ergänzende Einnahmequelle für das Gesundheitssystem dienen, insbesondere wenn alternative Finanzierungsformen ausgereizt sind. Drittens werden sie politisch häufig als Ausdruck individueller Eigenverantwortung begründet. Diese Funktionsvielfalt ist nicht unproblematisch. Wenn Zuzahlungen primär eine Finanzierungsfunktion erfüllen sollen, müssen sie hoch genug ausfallen, um relevante Einnahmen zu generieren, und werden damit schnell zur Zugangsbarriere. Wenn sie gleichzeitig steuern sollen, können Steuerungs- und Finanzierungslogik in Konflikt geraten. Im Folgenden werden Zuzahlungen daher konsequent als Steuerungsinstrument betrachtet. Aus dieser Perspektive lassen sich aus dem Ländervergleich 3 Bedingungen für ihre wirksame und gerechte Ausgestaltung ableiten.

Erstens ist die Steuerungswirkung von Zuzahlungen leistungsabhängig und zugleich nur begrenzt zielgenau. Über die untersuchten Länder hinweg zeigt sich ein konsistentes Muster: Zuzahlungen wirken im Arzneimittelbereich verlässlich und führen zu einer Reduktion in der Inanspruchnahme [43, 44]. Die preisliche Steuerung differenziert dabei jedoch nur begrenzt zwischen medizinisch notwendiger und nicht notwendiger Inanspruchnahme. Beispielsweise zeigen Chandra et al., dass höhere Zuzahlungen für Arztbesuche und Arzneimittel zwar zu einer reduzierten Inanspruchnahme dieser Leistungen führten, die Einsparungen jedoch durch Mehrausgaben im stationären Bereich teilweise kompensiert wurden („offset effect“; [53]). Bei chronisch Erkrankten war dies in einem Ausmaß der Fall, das die fiskalische Wirkung der Zuzahlungen weitgehend neutralisierte.

In der ambulant-ärztlichen Versorgung hingegen ist die Steuerungswirkung deutlich heterogener und stark von Ausgestaltung und institutionellem Kontext abhängig. Die deutsche Praxisgebühr blieb in ihrer Steuerungsfunktion weitgehend wirkungslos [24]. Ein internationaler systematischer Review zeigt zwar, dass Zuzahlungen für Hausarztbesuche in vielen Ländern zu einer reduzierten Inanspruchnahme führen, weist aber zugleich auf die erhebliche Variation der Effekte je nach Versichertengruppe und Ausgestaltung hin und bestätigt, dass wie bei Arzneimitteln die preisliche Steuerung nicht zwischen notwendiger und nicht notwendiger Versorgung differenziert [54]. Der Mechanismus dahinter ist plausibel und deckt sich mit den Erkenntnissen zu preiselastischer Nachfragesteuerung der HIE-Studie: Bei diskretionären Leistungen mit klarer Substitutionsmöglichkeit reagieren Versicherte auf Preise, bei der Grundversorgung dominieren Symptomdruck und ärztliche Empfehlung. Zuzahlungen können also nur dort steuern, wo Versicherte eine echte Wahl haben und die Inanspruchnahme von Leistungen nicht zwingend notwendig ist.

Zweitens entscheidet die Ausgestaltung über die Wirksamkeit. Gleiche nominale Beträge entfalten unterschiedliche Verhaltenseffekte je nach Konstruktion. Der Übergang vom Rückerstattungs- zum Selbstbehaltsmodell in den Niederlanden führte zu einer messbar stärkeren Reaktion der Versicherten [9, 31]. Verhaltensökonomisch ist dies durch die Verlustaversion erklärbar: Versicherte reagieren auf Verluste stärker als auf Gewinne in gleicher Höhe [55] und Selbstbehalte werden als unmittelbarer Verlust wahrgenommen, während Rückerstattungen oder Boni als hypothetischer, später anfallender Gewinn erscheinen. Hinzu kommt die Frage der Salience, also der Wahrnehmbarkeit der Zuzahlung im Moment der Entscheidung über eine Inanspruchnahme. Die deutsche Praxisgebühr war als pauschale Quartalszahlung kaum mit der konkreten Inanspruchnahme verknüpft, sodass das preisliche Signal im Versorgungsalltag verloren ging. Diese Mechanismen führen zu einem Zielkonflikt in der Ausgestaltung: Konstruktionen, die als Bonus oder Prämienrückerstattung gestaltet sind, erfahren höhere Akzeptanz selbst bei negativem Verlauf, entfalten aber eine geringere Steuerungswirkung. Konstruktionen mit unmittelbar fälligen Selbstbehalten steuern stärker, sind aber politisch weitaus schwerer vermittelbar [56]. Zuzahlungen entfalten ihre Steuerungswirkung folglich nur dann, wenn sie verhaltensökonomisch sinnvoll konstruiert sind, wobei es einen Zielkonflikt zwischen der Optimierung von Steuerungswirkung und Akzeptanz gibt.

Drittens entscheidet die institutionelle Einbettung über das Ergebnis. Frankreich zeigt, was geschieht, wenn die Steuerungslogik durch eine parallele Versicherungsstruktur neutralisiert wird. Die nominal hohen prozentualen Zuzahlungen werden für rund 95 % der Bevölkerung durch Zusatzversicherungen (*Mutuelles*) übernommen, sodass die Steuerungswirkung am *Point of Servic*e für die meisten Versicherten faktisch entfällt [10]. Was bleibt, ist eine Kostenverschiebung zwischen Versicherern, nicht aber eine Verhaltensänderung der Versicherten. Dänemark steht für einen anderen Pfad. Die preisliche Nachfragesteuerung in der ambulanten Regelversorgung wird durch ein striktes hausärztliches Gatekeeping institutionell ersetzt: Hausärzt:innen sind verbindliche Eintrittsstellen und überweisen in den spezialfachärztlichen und Krankenhausbereich [12]. Die institutionelle Architektur entscheidet damit, ob ein nominal vorhandenes Instrument seine Steuerungsfunktion überhaupt entfalten kann.

Quer zu diesen 3 Befunden steht die Frage, wer die finanziellen Lasten trägt. Die internationale Evidenz ist hier eindeutig: Uniforme Zuzahlungen wirken finanziell regressiv, d. h. belasten Personen mit geringem Einkommen überdurchschnittlich. Eine schwedische Studie auf Basis eines Registerdatensatzes zeigt, dass Zuzahlungen die ärztliche Inanspruchnahme im Durchschnitt um 5–10 % reduzieren. Der Effekt wird jedoch durch das untere Einkommensquartil getrieben, während Versicherte im oberen Quartil praktisch nicht reagieren. Diese Differenz lässt sich nicht durch Bildung, Familienstruktur oder Erwerbsstatus erklären; sie spiegelt eine tatsächliche Einkommensabhängigkeit der Reaktion wider [57]. Auf Systemebene zeigt sich diese Logik in Frankreich besonders deutlich: Während im Bevölkerungsdurchschnitt 2,1 % der Haushalte katastrophale Gesundheitsausgaben^1^ tragen, sind es im ärmsten Quintil 9 %; nahezu 90 % aller von katastrophalen Gesundheitsausgaben betroffenen Haushalte gehören der niedrigsten Einkommensgruppe an [37]. Die im Ländervergleich beobachteten Schutzmechanismen, z. B. Belastungsobergrenzen und krankheitsspezifische oder altersbedingte Befreiungen, adressieren dieses Verteilungsproblem in unterschiedlicher Logik und mit unterschiedlichem Erfolg. Folglich würde eine Implementierung von Zuzahlungen ohne adäquate bedarfs- und einkommensgerechte Schutzmechanismen zwar eine bedingte Steuerungswirkung entfalten, zugleich jedoch Personen mit geringen Einkommen überproportional belasten.

### Übertragbarkeit auf Deutschland.

In der deutschen Reformdebatte wird die Wiedereinführung einer Zuzahlung für Arztbesuche regelmäßig erwogen, zuletzt im Zusammenhang mit der Diskussion um ein Primärarztsystem. Die hier zusammengetragenen Befunde sprechen gegen eine Wiedereinführung der Praxisgebühr in ihrer früheren Form, aber für eine grundlegende Neukonzeption. Eine Zuzahlung als Generalinstrument zur Mengensteuerung der ambulanten Regelversorgung ist nach internationaler Evidenz wenig wirksam und die deutsche Erfahrung bestätigt dies. Sinnvoll erscheint stattdessen eine Konstruktion, die Zuzahlungen als gezieltes Steuerungselement im Rahmen einer koordinierenden Versorgungsstruktur einsetzt. Konkret könnte eine Zuzahlung dann fällig werden, wenn Versicherte den koordinierten Versorgungspfad verlassen, also z. B. direkt eine:n Spezialfachärzt:in aufsuchen oder ohne telefonische Triage die Notaufnahme in Anspruch nehmen. Damit wäre die Zuzahlung keine Zugangshürde zur Regelversorgung, sondern ein verhaltensökonomischer Anreiz an genau der Stelle, an der Fehlinanspruchnahme empirisch festgestellt wurde. Die konkrete Ausgestaltung eines Primärarztsystems ist dabei nicht Gegenstand dieses Beitrags.

Aus den Befunden ergeben sich 2 weitere Anforderungen an die Ausgestaltung eines Zuzahlungsmechanismus. Zum einen muss die Zuzahlung verhaltensökonomisch wirksam konstruiert sein. Eine Ex-ante-Information durch die Krankenkasse, beispielsweise eine jährliche Mitteilung über die zu erwartenden Kosten bei Pfadabweichung, würde die Salience erhöhen und der niederländischen Praxis der Krankenkassenrechnung beim *Eigen Risico* nahekommen. Zum anderen sollte eine Zuzahlung verteilungsgerecht konzipiert sein. Die Evidenz zur regressiven Wirkung uniformer Zuzahlungen legt nahe, eine einkommensbezogene Ausgestaltung in Erwägung zu ziehen. Eine vergleichbare Logik liegt auch jüngeren Reformvorschlägen für das französische System zugrunde, in denen eine einkommensbezogene jährliche Belastungsobergrenze für die gesamte Bevölkerung empfohlen wird [37]. Die konkrete Operationalisierung bedarf weiterer Untersuchung. Als Zusammenfassung für die laufende Reformdiskussion lässt sich festhalten: Eine Zuzahlung, die das Steuerungsproblem adressiert, ohne dabei neue Verteilungsprobleme zu schaffen, sollte einkommensbezogen, salient und auf die Pfadabweichung in einer koordinierten Versorgungsarchitektur fokussiert sein.

### Limitationen und Forschungsbedarf.

Die Evidenzbasis zu gesundheitlichen Outcomes von Zuzahlungen ist begrenzt. Ein systematischer Review weist auf reduzierte Inanspruchnahme präventiver Leistungen hin [58], belastbare Aussagen über langfristige Auswirkungen auf Morbidität und Mortalität sind auf der vorhandenen Evidenz jedoch kaum möglich. Methodisch beschränken sich vorhandene Studien meist auf quasiexperimentelle Auswertungen einzelner Reformen; vergleichende Untersuchungen zu Zuzahlungen innerhalb koordinierter Versorgungsarchitekturen sowie zu einkommensbezogenen Ausgestaltungen fehlen weitgehend. Hier liegt ein zentrales Forschungsfeld, dessen Bearbeitung eine evidenzbasierte Weiterentwicklung der nachfrageseitigen Steuerung in der GKV ermöglichen würde.

## Fazit

Der vorliegende Beitrag hat finanzielle Steuerungsinstrumente in 4 europäischen Gesundheitssystemen vergleichend analysiert. Eine pauschale Antwort auf die Frage der Wirksamkeit von Zuzahlungen ist empirisch nicht möglich. Wirksamkeit ist vielmehr eine Funktion dreier ineinandergreifender Bedingungen: der Leistungsart, der Berücksichtigung verhaltensökonomischer Effekte und der institutionellen Architektur. Für die deutsche Reformdebatte folgt daraus, dass eine Wiedereinführung der Praxisgebühr in ihrer früheren Form nicht zu empfehlen ist. Sinnvoll erscheint hingegen eine Konstruktion, die Zuzahlungen nicht als Generalinstrument zur Mengenreduktion einsetzt, sondern als gezieltes Lenkungsinstrument an jenen Stellen, an denen Fehlinanspruchnahme empirisch festgestellt wurde, etwa beim unangemessenen Verlassen eines koordinierten Versorgungspfades, z. B. im Rahmen eines Primärarztsystems. Voraussetzung wäre eine verhaltensökonomisch wirksame und verteilungsgerechte Ausgestaltung. Werden Zuzahlungen als gezieltes Lenkungsinstrument in einer koordinierten Versorgungsarchitektur konzipiert, können sie zur Verbesserung der Patientensteuerung beitragen, ohne den Zugang zur Regelversorgung zu gefährden.

## Data Availability

Für die Studie wurden keine Datensätze erstellt oder analysiert. Eine Angabe zur Datenverfügbarkeit ist deshalb nicht notwendig.

## Footnotes

1 Als katastrophale Gesundheitsausgaben werden Zuzahlungen für Gesundheitsleistungen definiert, die mindestens 10 % der gesamten Haushaltsausgaben oder des Haushaltseinkommens betragen (vgl. Definition WHO).
